# Retracted: The Favorable Effect of Mesenchymal Stem Cell Treatment on the Antioxidant Protective Mechanism in the Corneal Epithelium and Renewal of Corneal Optical Properties Changed after Alkali Burns

**DOI:** 10.1155/2022/9854827

**Published:** 2022-05-11

**Authors:** Oxidative Medicine and Cellular Longevity

*Oxidative Medicine and Cellular Longevity* has retracted the article titled “The Favorable Effect of Mesenchymal Stem Cell Treatment on the Antioxidant Protective Mechanism in the Corneal Epithelium and Renewal of Corneal Optical Properties Changed after Alkali Burns” [1], due to concerns with the figures. The journal was contacted by a reader who identified that Figure 5(a) in [1] is duplicated with 5A in [2]. Additionally, Figure 5(c) in [1] is duplicated with the H2 panel of Figure 3(b) in a now retracted article [3]. Figures 6(b) and 6(e) in [1] are duplicated with Figures 7(f) and 7(h) in [4].

The authors were asked for clarification, but did not satisfactorily address the concerns of the Editorial Board. The article is therefore being retracted due to concerns regarding the reliability of the data. Authors Dr. Jitka Cejkova and Dr. Cestmir Cejka are deceased and Dr. Peter Trosan did not respond to these concerns. The remaining authors agree to the retraction.
